# Enhancing Brain Tumor Diagnosis with L-Net: A Novel Deep Learning Approach for MRI Image Segmentation and Classification

**DOI:** 10.3390/biomedicines12102388

**Published:** 2024-10-18

**Authors:** Lehel Dénes-Fazakas, Levente Kovács, György Eigner, László Szilágyi

**Affiliations:** 1 Physiological Controls Research Center, University Research and Innovation Center, Obuda University, 1034 Budapest, Hungary; denes-fazakas.lehel@uni-obuda.hu (L.D.-F.); kovacs@uni-obuda.hu (L.K.); eigner.gyorgy@uni-obuda.hu (G.E.); 2 Biomatics and Applied Artificial Intelligence Institute, John von Neumann Faculty of Informatics, Obuda University, 1034 Budapest, Hungary; 3 Doctoral School of Applied Informatics and Applied Mathematics, Obuda University, 1034 Budapest, Hungary; 4 Computational Intelligence Research Group, Sapientia Hungarian University of Transylvania, 547367 Târgu Mureș, Romania

**Keywords:** brain tumors, MRI, segmentation, classification, neural networks, L-net, U-net, convolutional neural network (CNN), glioma, meningioma, pituitary tumor, automated diagnosis, medical imaging, deep learning

## Abstract

**Background:** Brain tumors are highly complex, making their detection and classification a significant challenge in modern medical diagnostics. The accurate segmentation and classification of brain tumors from MRI images are crucial for effective treatment planning. This study aims to develop an advanced neural network architecture that addresses these challenges. **Methods:** We propose L-net, a novel architecture combining U-net for tumor boundary segmentation and a convolutional neural network (CNN) for tumor classification. These two units are coupled such a way that the CNN classifies the MRI images based on the features extracted by the U-net while segmenting the tumor, instead of relying on the original input images. The model is trained on a dataset of 3064 high-resolution MRI images, encompassing gliomas, meningiomas, and pituitary tumors, ensuring robust performance across different tumor types. **Results:** L-net achieved a classification accuracy of up to 99.6%, surpassing existing models in both segmentation and classification tasks. The model demonstrated effectiveness even with lower image resolutions, making it suitable for diverse clinical settings. **Conclusions:** The proposed L-net model provides an accurate and unified approach to brain tumor segmentation and classification. Its enhanced performance contributes to more reliable and precise diagnosis, supporting early detection and treatment in clinical applications.

## 1. Introduction

Every year, hundreds of thousands of individuals receive a brain tumor diagnosis, with the United States alone recording approximately 18,000 fatal cases annually [1]. The likelihood of survival significantly improves with early detection. Nonetheless, factors such as the specific tumor type also play a pivotal role [2]. Detecting tumors in their early stages does not necessarily require precise tumor segmentation; rather, it is crucial that the detection process is sensitive enough to consistently identify even the smallest brain lesions. Brain tumors can exhibit a vast array of sizes, shapes, positions, and visual characteristics, making detection challenging due to deformations in normal brain structures and various forms of noise interference [3].

Over the past decade, there has been a remarkable surge in the development of methods for segmenting brain tumors, a surge largely catalyzed and sustained by the brain tumor segmentation (BraTS) Challenge, an annual event initiated in 2012 [4,5]. In the initial years of the BraTS initiative, researchers explored the entire classical spectrum of machine learning theories and practices [6]. More recently, the landscape of medical image analysis has witnessed a profound transformation, with deep learning techniques and an array of convolutional neural networks (CNNs) emerging as the predominant forces in this domain [7,8]. This paradigm shift underscores the remarkable progress and ongoing evolution in brain tumor segmentation methodologies.

While the brain tumor segmentation (BraTS) challenge primarily focused on gliomas, it is imperative to recognize the existence of diverse brain tumor lesions that necessitate precise classification for the establishment of a comprehensive brain tumor diagnostic pipeline. Consequently, the brain tumor segmentation problem can be regarded as an offshoot of the BraTS challenges, which has gained significant momentum in recent years. Numerous cutting-edge solutions, predominantly based on convolutional neural networks (CNNs), have emerged, boasting remarkable achievements, often attaining an accuracy of 97–98% in the challenging three-class brain tumor classification problem.

These state-of-the-art approaches not only leverage deep learning and convolutional networks but also incorporate various advanced techniques: Neelima et al. [9] introduced generative adversarial networks (GANs) into the domain of brain tumor segmentation. Kanchanamala et al. [10] proposed a deep hybrid representation learning approach, further enhancing the accuracy of brain tumor classification. Rajeev et al. [11] combined deep learning with Gabor wavelets to address brain tumor segmentation challenges. Mishra et al. [12] and Reddy et al. [13] introduced adaptive attention mechanisms to improve the segmentation of brain tumors. Mehnatkesh et al. [14] proposed a deep residual learning framework for robust brain tumor segmentation. Vankdothu et al. [15] explored recurrent CNNs to address the intricacies of brain tumor segmentation. Rahman et al. [16] introduced parallel deep CNNs as a promising approach to enhance brain tumor segmentation. Isunuri et al. [17] devised a multi-path convolution and multi-head attention network for accurate brain tumor segmentation.

These recent advancements underline the dynamic and rapidly evolving landscape of brain tumor segmentation research, bringing us closer to more accurate and effective diagnostic pipelines.

### Baseline and Background

The starting point of this work is represented by a previous study [18], where we deployed two different CNN networks based on VGG with a modified architecture, consisting of 14 and 17 layers, respectively. The main modification consisted of using MaxPooling layers with a 4×4 kernel. The convolution layers were Conv2D ones, with a kernel size of 3×3 in most of the cases, but we also studied versions with a 9×9 kernel in the first convolution layer. Batch normalization and MaxPooling were used after each convolution layer. The convolution blocks were followed by a flatten layer and three dense layers, where the last one performed the classification. The larger architecture also included a dropout layer placed in front of the dense layers, with a dropout rate set to 0.2. The number of filters in the first convolutional layer represented a relevant hyperparameter, whose value was chosen from the set {64,128,256,512}. The input image size was also used as a hyperparameter; we performed tests with image sizes of 128×128, 256×256 and 512×512. The number of neurons in the first two dense layers was set equal, and we tested network variants with 32, 256, 1024, 2048 and 4096 neurons in both layers. Also, we deployed two different learning strategies. Those strategies concerned the condition under which we save the weights of the model during learning. In one case, the strategy was to save for the smallest possible cost function. In the other case, it was if we increased the precision value on the TRAINING set by observing that metric during training. Tests were performed according to the five-fold cross-validation technique, meaning that the available data were divided into five equal groups, and each group took its turn to serve as testing data while the other four groups were used for training the chosen model. The average and standard deviation of the five runs were extracted. Since all available images received a prediction during the five runs, we also evaluated the network versions based of the sum of the five confusion matrices. The same five groups of images were used for all networks. In addition running thorough tests using various hyperparameter settings, we compared the classification outcome with state-of-the-art models like VGG with 16 layers and Resnet with 50 and 30 layers. Based on these tests, we proved that our architectures were able to be better adjusted to this classification problem than the state-of-the-art models. Our best achieved global accuracy was 98.27%, with a standard deviation of 0.66%. The best-performing model was running with 256×256 images. In addition, the neuron numbers in the dense layers were 256, the dropout layer was present, and the learning strategy was set to maximum accuracy.

In this study, we propose a more complex neural network to classify different brain tumors. Our solution was created by coupling a U-net and a CNN network. The data and the network architecture are discussed in detail in Section 2. We also report therein on our past results. In addition, we describe the teaching and testing methods we used. The metrics we use are also mentioned here. In Section 3, we describe our results with this more complex mesh structure. Finally, in Section 4, we summarize the results and set future plans.

## 2. Materials and Methods

### 2.1. Data

The dataset used for training and evaluating all CNN models involve in this study were originally compiled and made available for the public by Cheng et al. [19,20]. This dataset was meticulously assembled from 233 patients who received medical treatment at two distinct state-owned hospitals in Guangzhou and Tianjin, China, spanning the years 2005 to 2010. It comprises a total of 3064 T1-weighted contrast-enhanced brain MRI scans, each boasting a high resolution of 512×512 pixels per image, with a pixel size of 0.49 mm in both dimensions.

The dataset is rich and diverse, encompassing three distinct categories of brain tumors, specifically Pituitary, Meningioma, and Glioma, and covers three different image planes: axial, coronal, and sagittal views. These varied perspectives offer comprehensive insights into the structural characteristics and localization of these brain tumors. The dataset’s distribution is as follows: it contains 930 instances of Pituitary tumors, 708 instances of Meningioma tumors, and 1426 instances of Glioma tumors, ensuring a balanced representation of these different brain tumor types.

Each image in the dataset is provided in MATLAB (.mat) format, offering a comprehensive set of associated information. This includes a detailed description, a tumor mask delineating the tumor’s boundaries, a tumor class label indicating the specific tumor type, a tumor border to precisely demarcate the tumor region, and a unique patient identifier (ID) for traceability.

For a more comprehensive understanding of the dataset’s structural characteristics, Table 1 provides a detailed summary of its key attributes.

This dataset, with its rich and comprehensive attributes, serves as a valuable resource for the development and evaluation of brain tumor classification models, such as the L-net model proposed in the following.

### 2.2. U-Net: A Versatile Semantic Segmentation Framework

The U-net architecture, introduced by Ronneberger et al. in 2015 [21], is a deep learning model that has gained significant popularity in the field of semantic image segmentation. It was originally designed for biomedical image segmentation tasks, particularly for segmenting neuronal structures in microscopy images, but its versatility has led to its adoption in various domains beyond medical imaging.

#### 2.2.1. Architecture Overview

The U-net architecture derives its name from its distinctive U-shaped design. It consists of an encoder and a decoder, with a contracting path on the left side of the U and an expansive path on the right side.

The encoder, often referred to as the “downsampling” path, is responsible for capturing contextual information from the input image. It typically consists of multiple convolutional and pooling layers, which gradually reduce the spatial dimensions of the input while increasing the depth of feature representations.

The decoder, or the “upsampling” path, is responsible for generating the final segmentation mask. It consists of a series of up-convolutional (transposed convolutional) layers that gradually increase the spatial resolution of the feature map [22]. Skip connections are a key component of the U-net architecture, connecting corresponding layers in the encoder and decoder. These skip connections enable the model to retain fine-grained details from the input image.

#### 2.2.2. Skip Connections and Semantic Segmentation

The use of skip connections [23] in the U-net architecture is crucial for semantic segmentation tasks. These connections allow the decoder to access high-resolution feature maps from the encoder, which helps in preserving spatial information and capturing fine details. As a result, U-net excels at segmenting objects with well-defined boundaries in images, making it particularly suitable for tasks like medical image segmentation, where the precise delineation of structures is essential.

#### 2.2.3. Applications

The U-net architecture has found widespread use in various applications, including:

- Medical Image Segmentation: U-net has been extensively applied to segment various anatomical structures and abnormalities in medical images, such as tumors, organs, and blood vessels [24,25].
- Satellite Image Analysis: U-net is employed to segment land use, identify objects, and monitor environmental changes in satellite and aerial imagery [26,27,28].
- Industrial Quality Control: It is used for detecting and classifying defects in manufacturing processes, such as identifying defects in semiconductor wafers [29,30].
- Autonomous Vehicles: U-net assists in road scene understanding, enabling autonomous vehicles to identify objects and navigate safely [31,32,33].
- Biomedical Image Analysis: Beyond neuronal structures, U-net has been applied to segment cell nuclei, histological structures, and various biological objects [34].

The adaptability and effectiveness of the U-net in diverse domains have made it a go-to architecture for semantic segmentation tasks, and it continues to be a subject of research and innovation in the field of deep learning.

### 2.3. L-Net: U-Net Plus CNN

**Figure 1:**
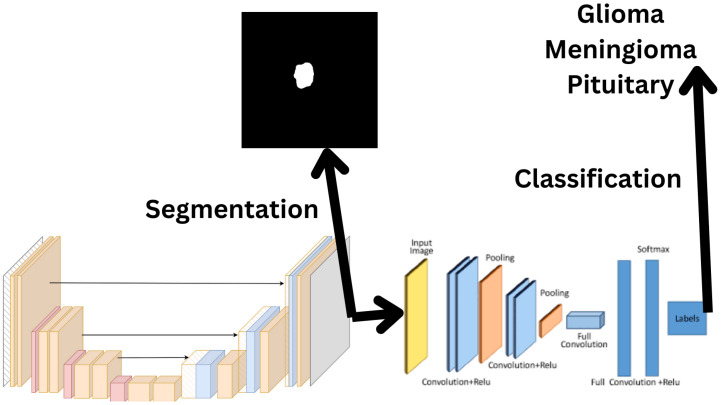
An overview of the L-net architecture, combining segmentation and classification tasks. The model first segments the tumor region using a U-net structure (**left**), where the black image represents the segmentation mask as the output. It then classifies the tumor, based on the features extracted by the U-net, into one of three categories: Glioma, Meningioma, or Pituitary (**right**), using a CNN.

In our current study, we propose a complex model, depicted in Figure 1, which is based on the observation that neural networks can learn better when trained on multiple problems simultaneously. The architecture consists of a U-net and a CNN connected in a special cascade. The input MR image is presented to the U-net, which provides at its output a predicted brain mask. The result provided by the U-net at the penultimate layer (just before the output layer) is fed to the input of the CNN instead of the original image, and thus the CNN predicts the tumor type from the information received from the U-net. The U-net is trained to predict segmentation result, based on the available tumor masks. The second part of the cascade, namely the CNN, is trained the provide an accurate prediction of the tumor type using the available annotation of images. The training of the two parts is performed simultaneously, not one after the other, so that each batch of images in each training epoch changes the weights in both parts of the architecture. The figures we use are similar to other manuscripts we have already written, since we have already dealt with segmentation and classification several times. However, they have been adapted to the current parameters used in the research [18,35,36,37].

#### 2.3.1. The U-Net Part of the L-Net

**Figure 2:**
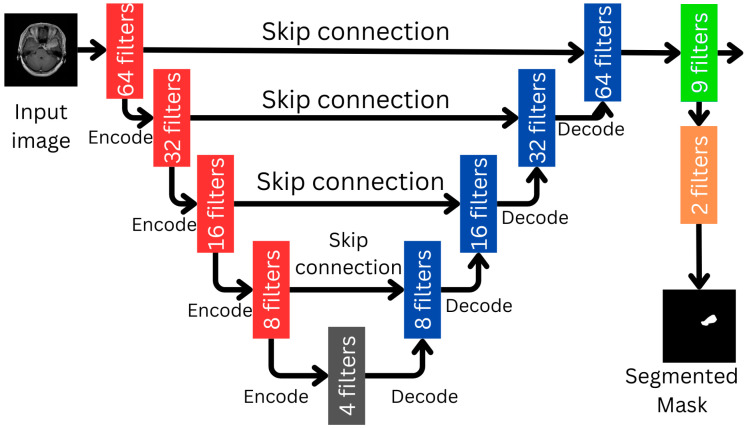
The U-net module, which forms a key part of the overall L-net architecture.

Our U-net, shown in Figure 2, contains four encoder blocks and four decoder blocks. These blocks are connected at the bottom by a bridge, as well as the skip connection at each level. As a difference in comparison to the original U-net, our model contains an extra convolution block with nine filters right before the output layer consisting of two filters marked in green, whose two filters give the predicted segmentation outcome. The filter numbers for the encoder blocks are 64, 32, 16 and 8, respectively, while the decoder blocks have the same filter numbers in reversed order. The bridge contains the smallest number of four filters as this is the smallest representation within U-net. Image sizes are not indicated in Figure 2, since multiple image resolutions were involved in the evaluation process. Each encoder block halves the image size in both directions, while each decoder doubles it to return to the original size at the output of the U-net. The CNN part of the L-net is connected after the layer marked in green, and uses it as input.

**Figure 3:**
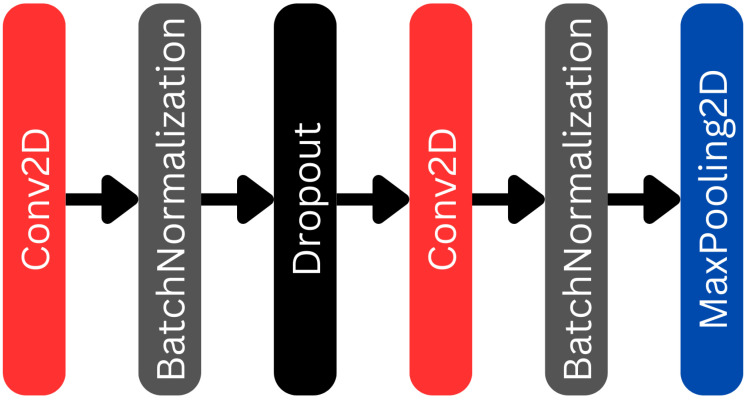
Encoder blocks of the U-net part of the L-net.

The structure of the encoder blocks is given in Figure 3, four such blocks are built in the encoder branch of the U-net network. These blocks consist of six layers, including two Conv2D layers, two batch normalization layers, a dropout layer, and a MaxPooling2D layer. The data progression within the block is shown in Figure 3. Data arriving to the block first pass through a Conv2D layer [38], whose output is fed into the batch normalization to obtain normalized data. After that comes a dropout layer that helps avoid overfitting [39]. The dropout rate is set to 0.2. Then comes the second Convd2D layer, whose output again is connected to a batch normalization layer. Finally, the MaxPooling2D [40] layer reduces the image dimensions. The matrix of the MaxPooling2D layer is 2×2, so the size of the images is halved compared to the input image. The skip connection obtains the output of the second batch normalization layer. That reduced-size output of the MaxPooling2D layer is wired into the subsequent decoder block. The Conv2D layers use an ELU [41] activation function. The filter numbers for each encoder block is indicated in Figure 2. The size of the convolution kernels is 3×3 and their fill factor is set. Thus, the size of the data does not change in the convolution layers.

**Figure 4:**
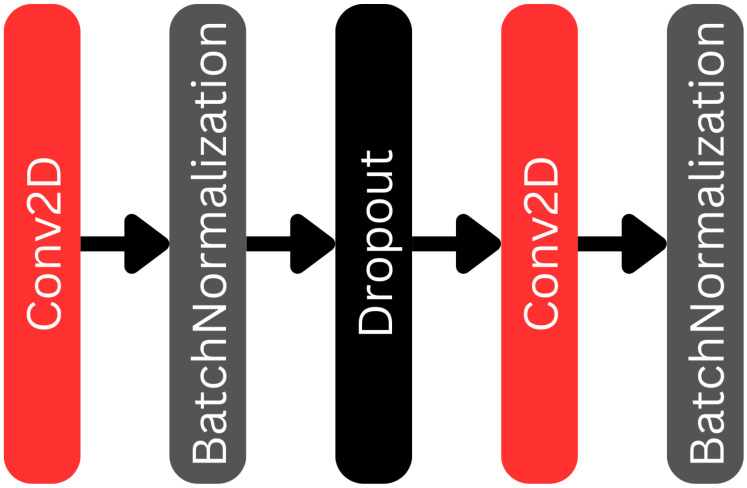
The bridge section within the U-net component of the L-net architecture.

The bridge part of our U-net network is shown in Figure 4. The bridge connects the encoder and decoder branches of the U-net network. Its structure is almost the same as the one of the encoder blocks, but there is no MaxPooling2D at the end of the bridge, and the image size is not reduced further.

**Figure 5:**
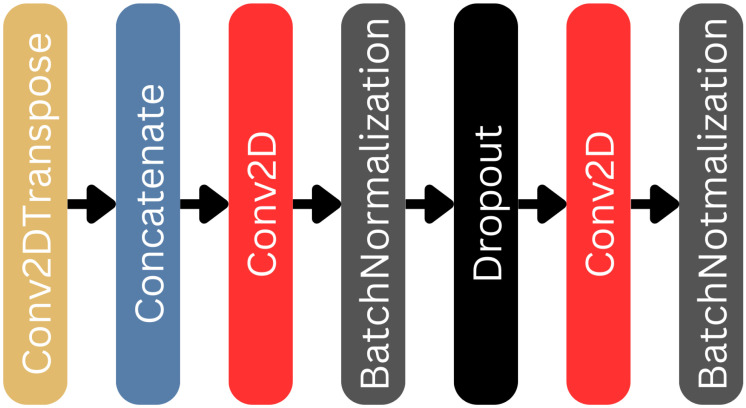
The decoder blocks within the U-net component of the L-net architecture.

The blocks of the decoder branch on the U-net part are built as indicated in Figure 5. These are the most complex blocks of the U-net, as they receive the output of the bridge or the output of the previous decoder, and after an upscaling of this lower-level data performed by the Conv2DTranspose layer, it is concatenated with the data received from the skip connection situated at the same level. The latter is performed by the Concatenate layer. The data then go through two Conv2D layers, each followed by a batch normalization, and with a dropout layer in between, with its dropout rate set to 0.2. The Conv2DTranspose layer uses a 2×2 kernel matrix, as well as a 2×2 stride, so it can double the size of the data it receives from the previous block. The filter number is the same as in the Conv2D layers situated within the given block. Similar to the encoder blocks, the Conv2D layers use kernels of size 3×3 and activation function ELU. The last decoder block outputs images that correspond in size with the original input of the U-net.

#### 2.3.2. The CNN Part of the L-Net

**Figure 6:**
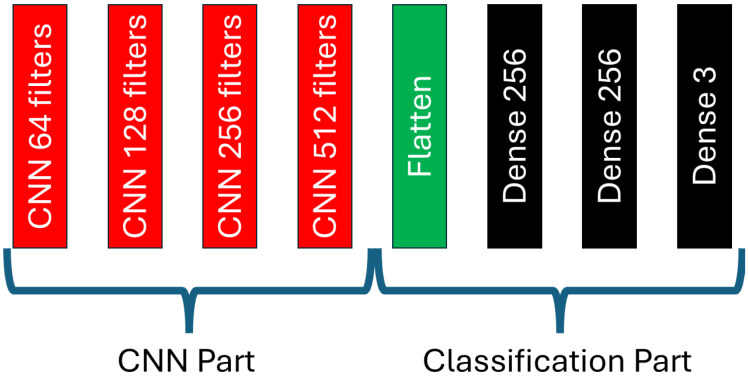
The detailed architecture of the CNN component that forms a crucial part of the overall L-net structure.

**Figure 7:**
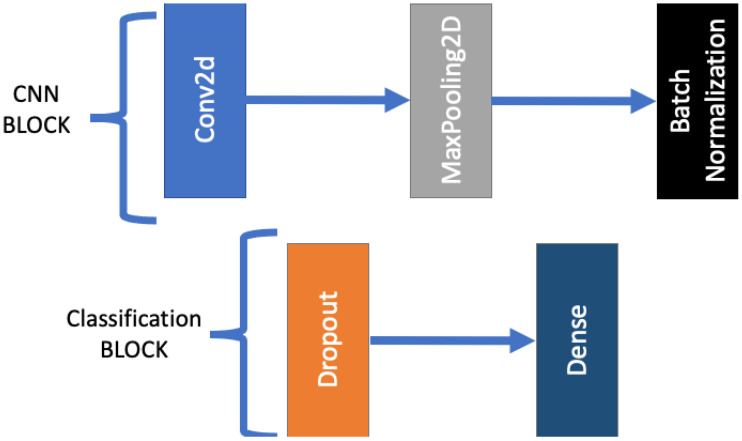
The individual blocks of the CNN component within the broader L-net architecture.

The convolutional part of the L-net architecture is responsible for classification. Its structure is shown in Figure 6. As already shown in Figure 1, the CNN is connected after the U-net. More precisely, Figure 2 indicates the exact connection point: the output of the penultimate layer of the U-net, consisting of nine filters, is fed to the input of the CNN. The CNN consists of a convolutional part responsible for feature extraction, and a classifier part which performs the actual classification. The blocks of the CNN network are shown in the Figure 7. The convolutional part consists of four convolutional blocks. The number of filters in the convolutional blocks are 64, 128, 256 and 512, respectively. The classifier part starts with a flatten layer that expands the elements in a column vector, which is then followed by three dense blocks. These dense blocks contain different number of neurons. The first two blocks contain 256 neurons, while the last one, which does the actual classification, contains three neurons corresponding to the three tumor classes that need to be distinguished.

These blocks, as mentioned above, come after the flatten layer, i.e., they only work with column vectors. The first layer of these blocks is the dropout layer with its rate set to 0.2, which was added to avoid overfitting. After that comes the dense layer, which represents a vector neuron set. The number of neurons per block is equal to the number of neurons in the corresponding layer, as shown in Figure 6. In addition, their activation function for this block is ReLU [42], with the exception of the last layer, which uses SoftMax activation [43] to predict probabilities for the actual classification.

### 2.4. Training and Testing

In this study we involved images in various resolutions, aiming to establish which resolution leads to the best classification accuracy. Due to the complexity of the proposed network, we had to limit the input image size. Training and testing sessions were separately performed at image resolutions of 16×16, 32×32, 64×64, and 128×128 pixels.

To ensure the fair circumstances for comparison with previous solutions, the training and testing was performed using the very same five groups of images described in Section 1. For each run of the evaluation process, training was performed in 1000 epochs using the Adam optimizer [44].

Our cost function was a complex one, since the network has two outputs. For segmentation, we employed a Dice loss function that we have successfully used in a previous work [36]. The essence of this cost function is to use sparse categorical cross-entropy (SCCE) [45], and, in addition, it computes the Dice score for each class. This Dice score is averaged and subtracted from 1. Then, weighted by 0.5, it is added to the SCCE cost, which is also weighted by 0.5. In the second output, the cost function was only SCCE. The total cost was the sum of these two cost functions. This was the basis on which our optimizer worked.

### 2.5. Performance Metrics

The performance of the classification methods used in this study is assessed through statistical indicators. Let us define Ψ={M,G,P} as the set of tumor classes, where M, G, and P correspond to the Meningioma, Glioma, and Pituitary tumors, respectively. The set of all images in class i∈Ψ, as identified by the ground truth, is denoted by $\Gamma_{i}$, while the set of images predicted to belong to the three classes is denoted by $\Lambda_{i}$, for any i∈Ψ. Let Γ represent the entire set of images used in the evaluation. Then, we have $\Gamma =\Gamma_{M}\cup \Gamma_{G}\cup \Gamma_{P}$ and also $\Gamma =\Lambda_{M}\cup \Lambda_{G}\cup \Lambda_{P}$.

Using the variables presented above, we can derive the following statistical metrics for any class i∈Ψ: sensitivity (also known as true positive rate, TPR, or recall), specificity (true negative rate, TNR), precision (positive predictive value, PPV), and the Dice similarity coefficient (DSC) or F1-score. Their formal definitions are provided in the upper part of Table 2. Additionally, the proportion of correct decisions across all tumor classes is referred to as accuracy (ACC), with its definition found in the lower part of Table 2.

Furthermore, we utilized the area under the receiver operating characteristic curve (AUC) as an additional benchmark to evaluate the classification performance.

All statistical accuracy indicators range between 0 and 1, with higher values indicating better accuracy.

### 2.6. Development Environments

During our research, we used the Python 3.8 environment, which was developed in the cloud using Google Colab. This Colab cloud service is useful because it has pre-installed libraries, which are important when building a neural network. The libraries that we used in the development—and can be found in Colab’s libraries—are Tensorflow 2.0.0 [46], Numpy [47], Scikit-learn [48] and Pandas [49]. Colab provided us with a Jupyter Notebook [50] environment for development, so the code was written in Jupyter Notebook. For our virtual environment, we also had resources provided by Colab. These resources are usually around 12 GB of video card memory. However, there is also the possibility of adding Google Drive, which is around 110 GB. We had 30 GB of RAM available. We also had four VCPUs.

## 3. Results and Discussion

The whole set of 3064 images was involved in a five-fold cross-validation process, using the very same five (almost) equal groups of images, which served as testing data in turns for the architecture trained on the other four groups of images. The proposed L-net model was evaluated with various hyperparameter settings and the classification outcome was compared with the baseline models. The segmentation results were not examined, as the L-net was working with a smaller image size than the original 512×512 resolution of the dataset. Under these circumstances, the segmentation results would be deceptive. Although the model provides a segmentation of the tumor in the input image, we focused only on the classification outcome, just like in our previous test described in Section 1. The results are exhibited in the following tables and figures.

Table 3 presents the precision, recall, F1 score and accuracy values obtained by the L-net model, and the mean and standard deviation of each metric obtained in the five folds of the cross-validation. In the case of the first three metrics, the average of the values obtained for the three different classes were averaged, and then the average and standard deviation of the five means values are presented in the table. In case of the accuracy, there is only one overall value in each fold which reflects the rate of correct decisions. The last row of the table also shows the best benchmarks obtained by the baseline model. One may notice that even with the lowest image resolution, the proposed L-net model provides a better classification outcome than the previous modified VGG model running at its optimal settings. The higher the image resolution, the better the obtained benchmarks, and the differences are relevant, as all benchmark values intensively tends towards 1 as the image resolution grows. We may also remark that the L-net models seems to be more stable than the previous VGG architecture, as the standard deviation of its accuracy indicators is much lower. At its best performance, L-net produces four times less misclassifications than the VGG model.

**Figure 8:**
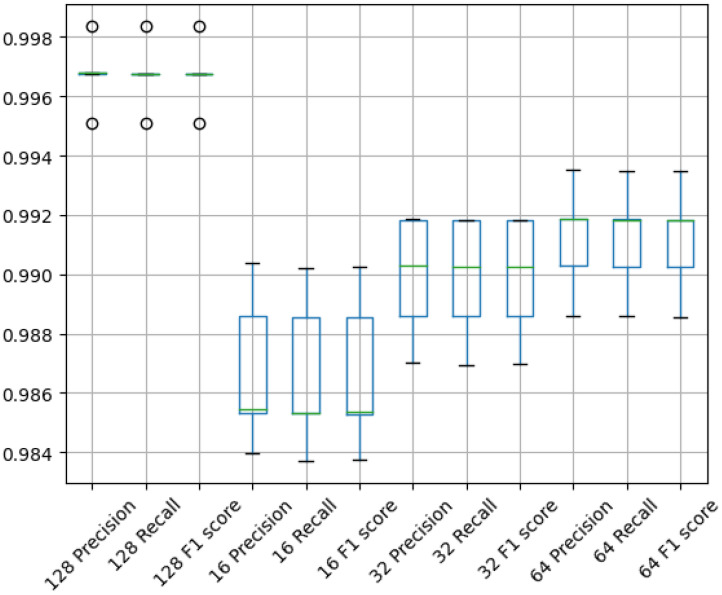
A boxplot for the precision, recall, and F1 score metrics. Lower axis indicates the image size and metric as well.

Figure 8 depicts the boxplots for the different metrics at various image sizes. As the image resolution increases, all observed benchmarks increase, making large steps towards 1. A step-like increase is observed: the median value rises and the inter quartile (IQR) range narrows. At the lowest resolution, L-net provides slightly better results than the previously modified VGG model’s best performance. As the image resolution doubles, the minimum value of benchmarks become higher or equal to the median obtained with the previous smaller resolution. As the resolution increases, the range between the quartiles gradually narrows. This trend results in a decreasing variance across the five test cases, indicating that the network’s generalization ability becomes increasingly stable. However, a notable and sudden decrease in the interquartile range occurs at the 128 resolution. This sharp decline suggests that, at this resolution, the network achieves a level of accuracy and consistency that significantly reduces the variability in test results. Consequently, there is less disparity between the tests, allowing for more reliable and precise outcomes. In other cases, there is already very little variance in the tests. Even in the 64 case, the variance is between 0.990 and 0.992. In the case of IQR 128 I show that three test cases are very close to each other around 0.997. Also, there are the two outliers below and above, which reach values below 0.996 and above 0.998.

Table 4 presents the AUC values obtained for each class of tumors, with the mean and standard deviation extracted from the five folds. Each row of the table represents a certain image resolution, and they are ordered in ascending order of the image size. In each case, it is obvious that very high AUC values are obtained for the Pituitary tumor class. Lower resolutions lead to AUC of 1. This proves that the Pituitary class is the one that the models can learn best, i.e., it is the least confused with other classes. It also has the lowest values for variance. Between the Meningioma and Glioma classes, there is no relevant difference in mean AUC values, as any of the two can be larger than the other in different rows. We can also observe that the AUC value of the Glioma class is obtained higher that the baseline model’s benchmark only at resolutions greater or equal to 64×64.

**Figure 9:**
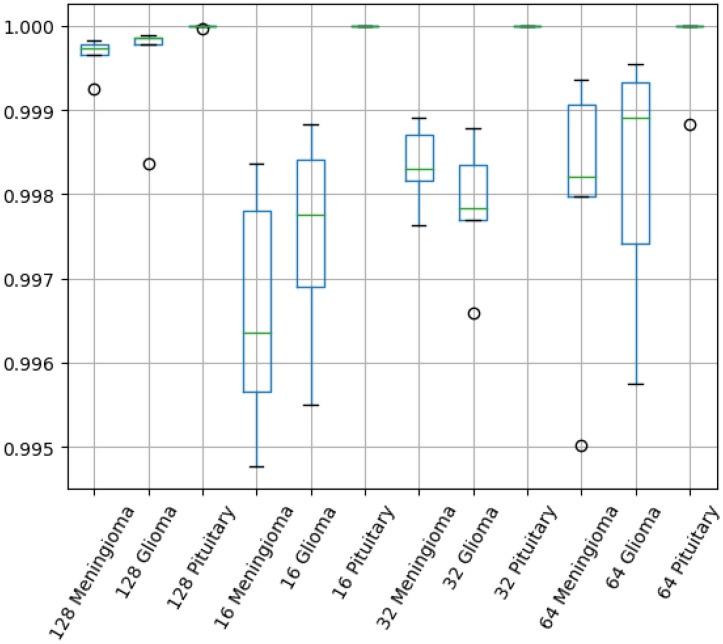
Boxplot for AUC metrics obtained for different tumor types and various image sizes.

Figure 9 exhibits the boxplots of AUC metrics listed in Table 4. The Pituitary class, in all test cases, can be seen to hover around an AUC of 1. This class can be easily distinguished by any other image class. In the case of the Glioma class, the lowest benchmarks are achieved at 16×16 resolution, with the minimum AUC value reaching 0.9955. The median value is close to but not reaching 0.998. Thus, it can be said that half of the tests fail to achieve an AUC value of 0.998 for Glioma classification at the lowest resolution. At an image size of 32×32 pixels, it is interesting to note that Glioma’s were less accurately detected than Meningioma tumors, which is not the case at other resolutions. At the 64×64 resolution, the median value rises to almost 0.999. However, its minimum is very close to the minimum reached in the smallest resolution case. At the highest tested resolution, namely 128×128, we can observe much higher AUC scores as the ones obtained at lower resolution, but there is an outlier among the five folds, affecting most the Glioma class and least the Pituitary tumor class.

Table 5 presents the overall confusion matrices obtained with the baseline VGG model using its four best-performing settings, and also those obtained by the L-net architecture at image sizes varying from 16×16 to 128×128. It can be easily noticed that the L-net model produces 40, 32, 20 and 10 misclassifications, respectively, in the four-image-size case, while the best baseline model made 53 mistakes while classifying the total number of 3064 tumor images. The number of correctly detected Meningioma cases achieved by the best set baseline model is equal to that obtained by L-net at 32×32 resolution, while at higher resolutions L-net makes less mistakes. In case of the other two classes, L-net deployed at any tested image resolutions correctly detects more tumors than the baseline model using any settings.

**Figure 10:**
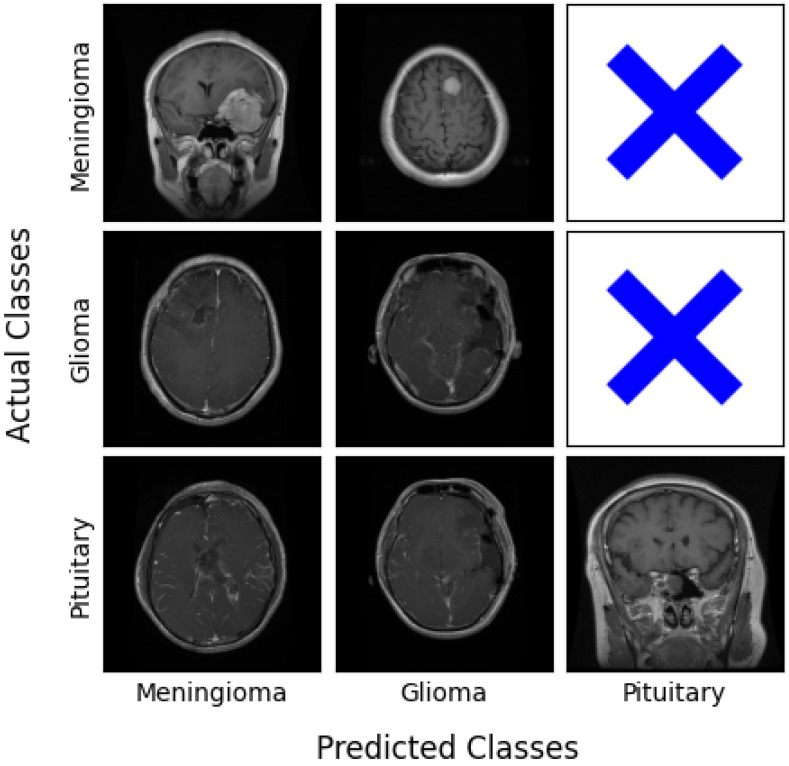
Some examples of correct and mistaken decisions made by the L-net architecture at the image size 128×128, organized in the format of a confusion matrix. The Pituitary class has no false positives.

Figure 10 shows some examples of input images, one for each case of the overall confusion matrix where available, reflecting the classification outcome of the best performing L-net network working at an 128×128 resolution.

Overall, we have proved that the idea can work, as employing a U-net to preprocess or to transform the input images into some intermediate data and feeding it to classification can greatly improve the accuracy of the decision. This is demonstrated by the previously presented evaluation results. Furthermore, we also proved that the proposed L-net model can work fine with even smaller images than a CNN-based classification. Table 3 shows the average results obtained with different metrics and their standard deviations. From the table, we can see that even for the 64×64 resolution, the average F1-score is higher than 0.99. Moreover, we can also see that even at the 16×16 resolution, we achieved better results than our previous solution. Additionally, it can be observed that the variance values are consistently decreasing with the proposed architecture. This decrease is well represented in Figure 8, which contains the boxplots. The higher the resolution, the smaller the variance of benchmarks, signifying that the learning process is much more stable. Table 4 gives the AUC metrics for each class. Very outstanding results are obtained for the Pituitary class. However, for this class, the best results are not obtained at the highest resolution of the input image. On the other hand, for the other classes, the 128×128 resolution performs the best. The most important results are represented by the confusion matrices; it is clear to see in Table 5 how much better the proposed L-net model classifies the images. The total number of misclassification reduced by up to 80%, in comparison to previous solutions. With our best L-net model, the Pituitary tumor class has no false positives at all, only a couple of false negatives. For the other two classes there are more misclassification, but still significantly less than in earlier solutions. The big step forward is clear, and we can assert that it makes sense to use a U-net to extract features for a CNN network model deployed for classification. The results of our method demonstrate that this network can be effectively used for tumor classification with high accuracy. Additionally, it offers the capability for segmentation. This network structure allows for the classification and detection of various types of diseases, particularly in cases where there may be multiple output classes within a segmentation region. For instance, the segmented region can include Glioma, Meningioma, and Pituitary tumors. However, to adapt the network for other datasets, retraining will be necessary, and modifications to the cost function may also be required.

## 4. Conclusions

In this paper, we proposed a novel architecture for the classification of MRI tumor images, which is performed simultaneously with a segmentation of the tumor. The proposed network has two main parts. First, a slightly modified U-net is deployed to segment the tumor from the input image, but the output of the penultimate layer in the U-net is used as an extracted feature, which is then fed to the second part of the architecture consisting of a modified VGG network. Compared to previous solutions, we achieved an improvement of classification accuracy from 98.3% to 99.6%, which represents a reduction to up to 80% of the misclassified cases. According to our previous findings, a classical VGG model best classifies these images at 256×256 resolution, while our model can surpass the accuracy of previous models even when using images at a 16×16 resolution. However, using a 128×128 image size is still recommendable, as it is beneficial with respect to the classification outcome. L-net is an excellent example to support the idea that training a neural network on two problems simultaneously can yield better results than performing the two works separately. In our case, the L-net learned both classification and segmentation. The results faithfully reflect that the error rate is now very low, as can be seen mainly from the confusion matrix. Also, the tables and boxplots show that the training is very stable at 128×128 resolution, with no large fluctuations. However, outliers occur for some metrics.

Further works will aim at the validation of the L-net architecture on various medical image datasets. This process presents the most difficulty, as few public image collections include both annotated segmentation and classification labels.

## Figures and Tables

**Table 1:** The structure of the image dataset involved in the study.

Tumor	Patient	Section Plane			Total
Type	Count	Coronal	Sagittal	Transversal	Images
Glioma	89	437	495	494	1426
Meningioma	82	268	231	209	708
Pituitary	62	319	320	291	930
Total	233	1024	1046	994	3064

**Table 2:** Accuracy indicators with respect to tumor class i∈Ψ (upper part), and overall accuracy (ower part).

Indicator	Definition
Sensitivity	${\mathrm{TPR}}_{i}=\frac{|\Gamma_{i}\cap \Lambda_{i}|}{|\Gamma_{i}|}$
Specificity	${\mathrm{TNR}}_{i}=\frac{|\left(\Gamma \setminus \Gamma_{i}\right)\cap \left(\Gamma \setminus \Lambda_{i}\right)|}{|\Gamma \setminus \Gamma_{i}|}$
Precision	${\mathrm{PPV}}_{i}=\frac{|\Gamma_{i}\cap \Lambda_{i}|}{|\Lambda_{i}|}$
F1 score	$F1_{i}=\frac{2\cdot |\Gamma_{i}\cap \Lambda_{i}|}{|\Gamma_{i}\left|+\right|\Lambda_{i}|}$
Accuracy	$\mathrm{ACC}=\frac{1}{\left|\Gamma \right|}\sum_{i\in \Psi }\left|\Gamma_{i}\cap \Lambda_{i}\right|$

**Table 3:** Precision and recall values obtained by the L-net model.

Image Size	Precision Mean ± Std	Recall Mean ± Std	F1 Score Mean ± Std	Accuracy Mean ± Std
16×16	0.986743 ± 0.002653	0.986622 ± 0.002665	0.986633 ± 0.002656	0.986622 ± 0.002665
32×32	0.989924 ± 0.002099	0.989884 ± 0.002121	0.989888 ± 0.002106	0.989884 ± 0.002121
64×64	0.991222 ± 0.001860	0.991189 ± 0.001853	0.991187 ± 0.001865	0.991189 ± 0.001853
128×128	0.996761 ± 0.001155	0.996737 ± 0.001153	0.996738 ± 0.001154	0.996737 ± 0.001153
Baseline	0.9817 ± 0.0068	0.9838 ± 0.0063	0.9827 ± 0.0066	0.9827 ± 0.0066

**Table 4:** AUC benchmarks obtained for different tumor classes.

Image Size	AUC Meningioma Mean ± Std	AUC Glioma Mean ± Std	AUC Pituitary Mean ± Std
16×16	0.996590 ± 0.001485	0.997482 ± 0.001324	1.000000 ± 0.000000
32×32	0.998341 ± 0.000495	0.997849 ± 0.000825	1.000000 ± 0.000000
64×64	0.997923 ± 0.001722	0.998192 ± 0.001595	0.999766 ± 0.000524
128×128	0.999646 ± 0.000229	0.999554 ± 0.000664	0.999992 ± 0.000017
Baseline	0.9961 ± 0.0008	0.9981 ± 0.0013	0.9987 ± 0.0013

**Table 5:** Overall confusion matrices obtained by the best four VGG models [18] (upper row), and the proposed L-net architecture at various image resolutions.

		Predicted			Predicted			Predicted			Predicted		
	Class	Meningioma	Glioma	Pituitary	Meningioma	Glioma	Pituitary	Meningioma	Glioma	Pituitary	Meningioma	Glioma	Pituitary
Actual	Meningioma	691	10	7	681	17	10	681	19	8	685	13	10
	Glioma	24	1399	3	25	1401	0	25	1401	0	29	1396	1
	Pituitary	5	4	921	3	2	925	3	2	925	3	3	924
		VGG first			VGG second			VGG third			VGG fourth		
		Predicted			Predicted			Predicted			Predicted		
	Class	Meningioma	Glioma	Pituitary	Meningioma	Glioma	Pituitary	Meningioma	Glioma	Pituitary	Meningioma	Glioma	Pituitary
Actual	Meningioma	683	21	4	691	12	5	695	9	4	704	4	0
	Glioma	12	1414	0	11	1415	0	5	1421	0	4	1422	0
	Pituitary	1	2	927	3	1	926	1	1	928	1	1	928
		16×16			32×32			64×64			128×128		

## Data Availability

Access to the data is available upon request. Access to the data can be requested via e-mail to the corresponding author.

## Abbreviations

The following abbreviations are used in this manuscript:

BraTS	Brain Tumor Segmentation
GANs	Generative Adversarial Networks
MRI	Magnetic resonance imaging
AI	Artificial Intelligence
CNN	Convolutional neural network
CNNs	Convolutional neural networks
ELU	Exponential Linear Unit
SCCE	Sparse categorical cross-entropy
TPR	Sensitivity or True Positive Rate
TNR	Specificity or True Negative Rate
PPV	Precision or Positive Predictive Value
DSC	Dice Similarity Score
ACC	Accuracy
